# Investigating automated regression models for estimating left ventricular ejection fraction levels in heart failure patients using circadian ECG features

**DOI:** 10.1371/journal.pone.0295653

**Published:** 2023-12-11

**Authors:** Sona M. Al Younis, Leontios J. Hadjileontiadis, Aamna M. Al Shehhi, Cesare Stefanini, Mohanad Alkhodari, Stergios Soulaidopoulos, Petros Arsenos, Ioannis Doundoulakis, Konstantinos A. Gatzoulis, Konstantinos Tsioufis, Ahsan H. Khandoker

**Affiliations:** 1 Department of Biomedical Engineering, Healthcare Engineering Innovation Centre (HEIC), Khalifa University, Abu Dhabi, United Arab Emirates; 2 Department of Electrical and Computer Engineering, Aristotle University of Thessaloniki, Thessaloniki, Greece; 3 Creative Engineering Design Lab at the BioRobotics Institute, Applied Experimental Sciences Scuola Superiore Sant’Anna, Pontedera (Pisa), Italy; 4 Cardiovascular Clinical Research Facility, Radcliffe Department of Medicine, University of Oxford, Oxford, United Kingdom; 5 First Cardiology Department, School of Medicine, “Hippokration” General Hospital, National and Kapodistrian University of Athens, Athens, Greece; Tehran University of Medical Sciences, ISLAMIC REPUBLIC OF IRAN

## Abstract

Heart Failure (HF) significantly impacts approximately 26 million people worldwide, causing disruptions in the normal functioning of their hearts. The estimation of left ventricular ejection fraction (LVEF) plays a crucial role in the diagnosis, risk stratification, treatment selection, and monitoring of heart failure. However, achieving a definitive assessment is challenging, necessitating the use of echocardiography. Electrocardiogram (ECG) is a relatively simple, quick to obtain, provides continuous monitoring of patient’s cardiac rhythm, and cost-effective procedure compared to echocardiography. In this study, we compare several regression models (support vector machine (SVM), extreme gradient boosting (XGBOOST), gaussian process regression (GPR) and decision tree) for the estimation of LVEF for three groups of HF patients at hourly intervals using 24-hour ECG recordings. Data from 303 HF patients with preserved, mid-range, or reduced LVEF were obtained from a multicentre cohort (American and Greek). ECG extracted features were used to train the different regression models in one-hour intervals. To enhance the best possible LVEF level estimations, hyperparameters tuning in nested loop approach was implemented (the outer loop divides the data into training and testing sets, while the inner loop further divides the training set into smaller sets for cross-validation). LVEF levels were best estimated using rational quadratic GPR and fine decision tree regression models with an average root mean square error (RMSE) of 3.83% and 3.42%, and correlation coefficients of 0.92 (p<0.01) and 0.91 (p<0.01), respectively. Furthermore, according to the experimental findings, the time periods of midnight-1 am, 8–9 am, and 10–11 pm demonstrated to be the lowest RMSE values between the actual and predicted LVEF levels. The findings could potentially lead to the development of an automated screening system for patients with coronary artery disease (CAD) by using the best measurement timings during their circadian cycles.

## Introduction

Heart failure (HF) affects approximately 26 million people worldwide [1], which is an increasing global burden on cardiologists with 3.5 million new patients yearly [2]. At 55 years of age, the lifetime risks of heart failure for men and women are 29% and 33%, respectively [3]. According to estimates, 480,000 adults (aged 18 or older) suffer from cardiac failure, which accounts for 2.1% of the population in adults [4]. While there are slight variations in the definitions of heart failure used in the current practice guidelines from the American College of Cardiology (ACC)/American Heart Association (AHA) [5], Heart Failure Association (HFA)/European Society of Cardiology (ESC) [6], and Japanese Heart Failure Society (JHFS) [7], the overall concepts and criteria for HF classification into different stages are based on the severity and progression of the condition and the identifiable signs and symptoms including edema/fluid retention, dyspnea, activity intolerance, and fatigue. They also emphasize the presence of structural or functional heart disease as a prerequisite for the diagnosis. Coronary artery disease (CAD) is the leading cause of HF, followed by hypertension, diabetes, valvular heart disease, and cardiomyopathy [8]. Left Ventricle Ejection Fraction (LVEF) levels, which is a hemodynamic term for the fraction of ventricular volume ejected per heartbeat [9], was further highlighted as important indicators for diagnosis, prognosis, and treatment of HF patients [10, 11], physical examination, patient history, and clinical tests [12].

According to the American Society of Echocardiography and the European Association of Cardiovascular Imaging (ASE/EACVI) [13, 14], systolic dysfunction, usually called heart failure with reduced ejection fraction (HFrEF), is the clinical manifestation of HF symptoms with a resulting LVEF of less than 50%. If the measured EF is more than 55% it is a diastolic dysfunction, usually called heart failure with preserved ejection fraction (HFpEF). Nevertheless, if LVEF is slightly reduced (LVEF 50–55%), the failure category is heart failure with mid-range ejection fraction (HFmEF). Due to the HF etiology, the HFmEF category’s narrower range is seen as a changeable criterion for this category. Different cut-off values for the classification of HF are advised by other standards, such as the ESC [6] and JHFS [7]; the cut-off for HFrEF is as low as 40%. According to the literature, there are no rigid guidelines, and the treatment is only tangentially related to LVEF and clinical presentation. However, based on the ESC criteria, patients in the mid-range group between 40 and 49% showed that 90% of patients either got better or got worse, with only 10% of instances remaining unaltered.

LVEF is estimated from nuclear medicine scans [15], computerized tomography (CT) [16], and cardiac catheterization [17]. However, cardiovascular magnetic resonance (CMR) [18] imaging and echocardiography [19] are the most reliable and widely used methods to evaluate LVEF. Despite the use of non-ionizing radiations and high estimation accuracy, potential limitations are associated with these techniques, i.e., cost and accessibility, operator dependency, patient contraindications, time and patient cooperation, and image interpretation challenges. Electrocardiography (ECG) presents a supplementary tool that is both accessible and cost-effective for the evaluation of LVEF. The choice of ECG as a method for LVEF assessment is substantiated by its capability to capture key physiological connections reflective of cardiac performance. ECG features, such as QRS duration, ST segment changes, and T-wave abnormalities, are indicative of electrical conduction abnormalities, myocardial ischemia, and ventricular repolarization anomalies. These electrical manifestations are intricately linked to the mechanical processes governing ventricular contraction and relaxation. Notably, alterations in LVEF can influence cardiac electrical activities, giving rise to deviations in ECG patterns. Consequently, by comprehensively analysing ECG signals and their derived parameters, it becomes plausible to discern the interplay between these electrical indicators and LVEF, offering valuable insights into cardiac function without the necessity of more invasive or costly procedures.

ECG is one of the most widely used non-invasive diagnostic tools for tracking the physiological activities of the heart throughout time. Many cardiovascular disorders, including atrial fibrillation, myocardial infarction, premature contractions of the ventricles or atria, and congestive heart failure, can be diagnosed with the help of ECG data [20–25]. Consequently, automatic and accurate ECG data analysis has become a popular study area, especially using artificial intelligence (AI) tools. The diagnostic golden rules have been used for automatic ECG analysis. This process consists of two steps that involve human specialists to create meaningfully featuring from raw ECG data; these features could be categorized into coefficients of variation and density histograms, statistical features (such as heart rate variability), frequency-domain features, time-domain features, and sample entropy. Based on ECG and machine learning tools, several researchers classified patients exhibiting congestive HF from normal [26, 27]. In contrast, deep learning with one lead ECG signal was used to categorize HF patients into CAD, Myocardial Infarction (MI), and congestive HF classes [20].

However, there is still a limited understanding of the intricate relationship between circadian ECG wave characteristics and the estimation of LVEF in patients with heart failure. Moreover, it would be valuable to present a more accessible alternative approach for evaluating LVEF that doesn’t necessitate extensive expertise or costly equipment. Along the same lines, machine learning techniques, including deep learning, can play a crucial role in comprehending the intricate ECG features present in-patient records, ultimately leading to improved assessment of HF. Hence, the objectives of this study were:

1. To explore the potential of machine learning regression models trained on features extracted from the ECG waveform.
2. In line with the ASE/EACVI guidelines, to accurately estimate LVEF levels in HFpEF, HFmEF, and HFrEF HF categories.
3. To analyse the cardiovascular system dynamics in HF patients hour by hour throughout a 24-hour day.
4. To emphasize the heart’s 24-hour circadian functionality (correlated with ECG features) to recommend the best times for a correct estimation of LVEF levels, offering a suitable screening strategy with the best window of possibilities for halting heart failure progression.

## Materials and methods

### Dataset and patients’ enrollment

**Fig 1:**
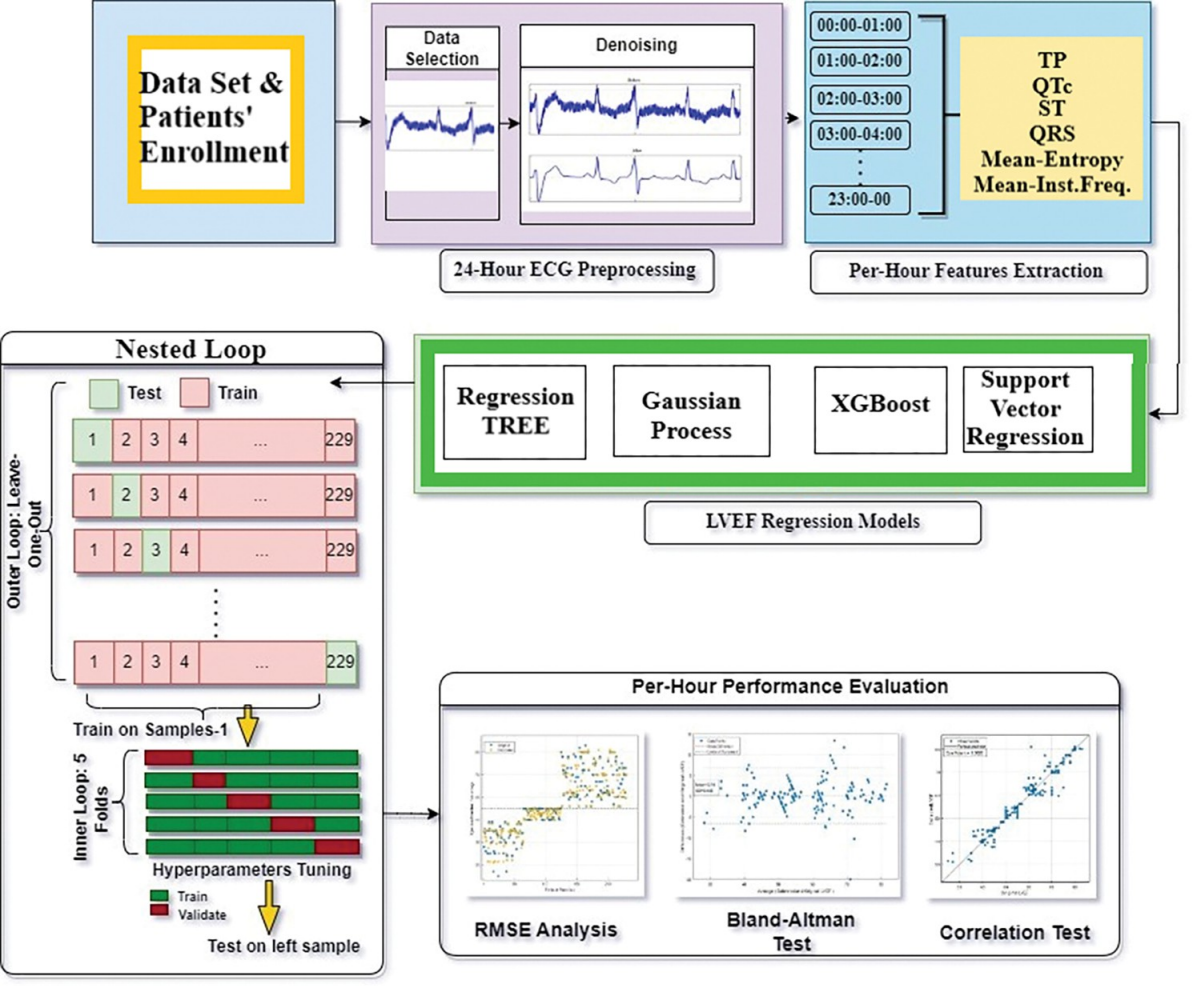
An illustration of the overall procedure followed in this study.

The complete procedure followed in this study is illustrated in Fig 1. This study used two datasets that comprised clinical data from patient groups of Greeks and Americans. Patients with HF, specifically CAD, and ages ranging from 33 to 88 years old (n = 303) were included in both datasets. According to the ASE/EACVI recommendations, these patients were split into 129 HFpEF, 92 HFmEF, and 82 HFrEF groups.

Patients from seven cardiology departments in Greece who were engaged in the PRESERVE EF trial contributed to the Greek patient cohort [28]. The enrolment protocol of patients for the PRESERVE EF study(clinicaltrials.govidentifier NCT02124018) was approved by the ethics committee at each of the seven selected cardiology departments at Greece and was endorsed by the Hellenic Society of Cardiology. The Hellenic Society of Cardiology established and maintained a database [29]. Each patient signed a consent form before enrolling in the trial at each cardiology department. Patients needed to meet the following requirements to be eligible for enrolment: (1) having a post-angiographically proven MI at least 40 days after the event or 90 days after any CABG surgeries, if applicable; (2) being revascularized; (3) not being revascularized but lacking evidence of any active ischemia in the previous six months; and (4) completing an effective course of medical treatment.

Additionally, any patient who had a secondary prevention indication for the placement of an implantable cardioverter defibrillator (ICD), a permanent pacemaker, persistent, long-standing persistent, or permanent atrial fibrillation, any neurological symptoms of syncope or pre-syncope within the previous six months, or the presence of any systemic illnesses like liver failure, renal diseases, rheumatic diseases, thyroid dysfunction, or cancer was excluded from the study. For all patients, the American Society of Echocardiography’s recommendations are followed when doing echocardiographic tests, and a GE Healthcare GETEMED CardioDay Holter system (recorder CardioMem CM4000 and software CardioDay v. 2.4, GE Healthcare, Fairfield, CT, USA) is used.

The Intercity Digital Electrocardiography (ECG) Alliance (IDEAL) study archives at the University of Rochester Medical Centre Telemetric and Holter ECG Warehouse (THEW) were used to identify the American patient cohort [30]. The database enrolment protocol complied with the Declaration of Helsinki and Title 45, U.S. Code of Federal Regulations, Part 46, Protection of Human Subjects (revised: November 13, 2001-effective: December 13, 2001). Additionally, the IDEAL protocol was authorised by the University of Rochester’s research subject review board [31]. Before taking part in the trial, each patient gave their written consent.

The following requirements had to be met to qualify for enrolment in the IDEAL study: (1) having evidence of either a prior MI or exercise-induced ischemia; (2) being in the stable phase of ischemic heart disease at least two months after the last event; (3) not having been given a congenital heart failure diagnosis; and (4) having sinus rhythm. Additionally, any patients with dilated cardiomyopathy—defined as having a left ventricular diameter (LVD) > 60 mm and an ejection fraction (EF) 40%—congenital heart failure (CHF), coronary artery bypass grafting (CABG) surgery, non-sinus rhythm, and any cerebral, severely hepatic, or malignant diseases—were disqualified from the trial. All patients underwent an echocardiography examination to determine their LVEF levels. In addition, a 24-hour ECG test was performed on each patient utilising the pseudo-orthogonal lead configurations (X, Y, and Z).

From eligible individuals, a 24-hour ECG Holter recording was obtained and sampled with 200 Hz. In this study, patients with missing recordings of an hour or more were excluded. Thus, the dataset included only 229 patients; split into 105 HFpEF, 60 HFmEF, and 64 HFrEF according to the ASE/EACVI guidelines. More details on the selected dataset are provided in Table 1.

**Table 1:** Clinical characteristics of the heart failure patients based on their LVEF categories.

Clinical Variables	Overall Subjects	LVEF Categories			p-value
		HFpEF (n =)	HFmEF (n =)	HFrEF (n =)	
**Patients (n)**	229	105	60	64	-
**LVEF (%)**	25–82	56–82	50–55	25–49	<0.001
**(Mean±Std)**	(56.16±11.79)	(66.63±6.7)	(52.82±2.24)	(42.12±5.52)	-
**Gender (M/F)**	196/33	90/15	49/11	57/7	0.237
**Age (yrs.)**	35–88	35–79	40–88	40–80	0.110
**(Mean±Std)**	(58.29±10.75)	(57.14±10.4)	(60.75±10.88)	(57.86±10.97)	-
BMI (kg/m^2^)	17.99–37.89	19.72–36.33	17.99–37.65	20.8–37.89	0.133
**(Mean±Std)**	(27.15±3.62)	(26.66±3.44)	(27.49±3.89)	(27.61±3.59)	-
**Smoking (Yes/No)**	167/62	72/33	48/12	47/17	0.757
**Diabetes (Yes/No)**	29/200	7/98	7/53	15/49	<0.001
**Hypertension (Yes/No)**	113/116	51/54	31/29	31/33	0.587
**Angina (Yes/No)**	159/70	83/22	37/23	39/25	0.189
**VT (Yes/No)**	19/210	9/96	2/58	8/56	0.088
**Prior-MI (Yes/No)**	150/79	54/51	44/16	52/12	<0.001
**Medications (Beta-Blockers, ACE Inhibitor, Anti-Arrhythmic, Diuretic)**					
**Beta-Blockers (Yes/No)**	179/50	80/25	46/14	53/11	0.478
**ACE Inhibitor (Yes/No)**	68/161	30/75	15/45	23/41	0.611
**Anti-Arrhythmic (Yes/No)**	7/222	3/102	4/56	0/64	0.166
**Diuretic (Yes/No)**	42/187	1/104	19/41	22/42	<0.001

BMI: Body Mass Index, VT: Ventricle Tachycardia, MI: Myocardial Infarction, ACE: Angiotensin converting enzyme.

### Pre-processing and feature extraction

The 24-hour circadian ECG was fixed to start at 12:00 am for all the patients using a cosinor ftting analysis [32]. The SDROM-ADF filter [33] was used to filter each hour of the Holter ECG data for noise. Time- and frequency-domain features were extracted from the denoised ECGs, as explained next. The P-QRS-T components of the ECG wave have been accurately analysed using a variety of intricate approaches. A typical ECG is composed of the P wave, QRS complex, and T wave. Electric currents created by the ventricles’ depolarization before to contraction, through the ventricular myocardium’s depolarization extension, result in the QRS complex. The P wave, on the other hand, is created by electrical currents that the atria depolarize before contracting. The related definition and description of the ECG features are provided in Table 2.

**Table 2:** Definitions of ECG features.

ECG Feature	Definition
**TP (ms)**	Signifying the period when the ventricles are repolarized and at rest. Variations in the TP interval may show abnormalities in repolarization, which can be linked to impaired cardiac function in HF patients.
**QT (ms)**	Reflects the total time for ventricular electrical activity, including both depolarization and repolarization. It is influenced by heart rate and can be used to assess the risk of arrhythmias.
**ST (ms)**	Reflects the period when the ventricles are electrically inactive and transitioning from depolarization to repolarization. Deviations in the ST segment can indicate myocardial ischemia or injury.
**QRS (ms)**	Represents the contraction of the ventricles, responsible for pumping blood to the systemic and pulmonary circulation.
**Mean-Entropy**	Used to analyse the complexity and unpredictability of the ECG. Higher mean entropy might indicate more irregular and complex patterns, which could be linked to various conditions or changes in the cardiac function.
**Mean-Instant Frequency**	A measure of how rapidly the frequency of a signal is changing at a particular point in time. Changes in instant frequency could relate to heart rate variability or arrhythmias.

The QT interval varies significantly according to gender, age, heart rate, and drug use. As a result, the QT interval can be calculated using Bazett’s method while taking variations into account [34]. RR refers for the R-R interval, while QTc (QTc = QT/RR) stands for the QT interval corrected. The Slope Intersect (SI) approach was used in this study’s automatic QT interval detection [35]. The Pan Tompkins ECG QRS detector was applied in Matlab to find the QRS complex [36]. Furthermore, TP, PR, and ST-T were extracted using the ECG Matlab Toolbox [37]. The power spectrogram’s initial moment was used to estimate the instantaneous frequency of the signal using 255-time frames. In addition, the spectral entropy of the power spectrogram was calculated to gauge how flat and spiky a signal’s spectrum is.

### Machine learning and training settings

To provide an effective regression process, it is crucial to have the ability to train and estimate LVEF levels in HF patients using machine learning algorithms. In this study, four models were used, including Support Vector Machine (SVM), Extreme Gradient Boosting (XGBOOST), Gaussian Process Regression (GPR), and Decision Tree Regression (TREE), to assess the effectiveness of various machine learning methods.

### 1) SVM

A standard and adaptable machine learning method is often used for classification problems. However, Support Vector Regression (SVR), a variation of SVM, can be utilised for regression tasks. It manages interactions between the input characteristics and the target variable that are both linear and nonlinear. It captures complicated patterns and nonlinearity by mapping the data into a higher-dimensional space using a kernel function [38]. SVR is less sensitive to outliers compared to some other regression techniques.

Finding a "tube" or margin around the best-fitting function is the fundamental tenet of SVR; data points inside the tube are regarded as well-fitted, while those outside the tube are penalised. Using a margin around the regression line allows for some error tolerance leading to robustness to outliers.

SVM regression has only a few tuning parameters, such as the choice of kernel and the regularization parameter, making it easy to use and implement [39]. The two main hyperparameters to tune are the kernel and the regularization parameter;

1. Kernel Selection: SVRs can capture nonlinear relationships since it uses a kernel function to translate the input into a higher-dimensional space. The SVR model’s performance can be considerably impacted by kernel selection. Typical kernels include linear kernel, polynomial kernel, and radial basis function (RBF).

2. Regularization Parameter (C): The regularization parameter (C) determines the trade-off between maximizing the margin and minimizing the training error. A smaller C allows a larger margin but may lead to more training errors, while a larger C leads to fewer errors but may result in a smaller margin.

In this work, the kernel types are selected to be Linear, Polynomial, and RBF, and we defined the C Values in the range of: [0.1, 1, 10,14].

### 2) XGBOOST

It is an ensemble learning technique that builds a powerful predictive model by combining several weak predictive models, often decision trees, commonly used to predict heart diseases [40, 41]. The XGBoost approach is a development of gradient boosting, adding decision trees to the model iteratively while each tree tries to fix the mistakes caused by the preceding one. However, XGBoost incorporates several enhancements to improve performance, speed, and generalization; to reduce overfitting and increase model generalisation, this model incorporates L1 (Lasso) and L2 (Ridge) regularisation terms. In addition, it employs a novel method to accelerate convergence while improving model accuracy by optimising the loss function during tree construction.

XGBoost provides a measure of feature importance, which helps find the most noteworthy features for making predictions. It can automatically handle missing data in the input features, making it more robust in real-world datasets. It also supports k-fold cross-validation, aiding in assessing model performance and hyperparameter tuning. Although many hyper-parameters are included in XGBoost, in this work, the hyper-parameters adopted for tuning include:

1) Learning Rate: The step size shrinkage used in the update to prevent overfitting. Lower values make the boosting process more conservative.

2) Maximum Depth: The maximum depth of a tree. Deeper trees can capture more complex patterns but may lead to overfitting.

2) Gamma: The minimum loss reduction required to partition a leaf node further. It acts as a regularization term, controlling the complexity of the trees and preventing overfitting.

The learning rate (eta) is set to be in the range of: [0.1, 0.2], the maximum depth values are: [3, 5], and the selected gamma values are: [0, 0.01]. We use the default settings in Matlab for the rest of the other hyperparameters.

### 3) GPR

A potent non-parametric probabilistic regression method for modelling and forecasting continuous data is called GPR. It is based on Gaussian processes [42], which are groups of randomly distributed variables with a common distribution. In GPR, the objective is to develop a distribution over potential functions that could explain the data to represent the link between input data and output values. In contrast to parametric regression models, which require learning specific model parameters, GPR predicts a distribution across functions consistent with the training data. A mean function and a covariance function, referred to as a kernel function, represent this distribution. The fundamental assumption in GPR is that nearby data points in the input space should have similar output values. The covariance function captures the similarity between data points and plays a crucial role in shaping the posterior distribution over functions. The choice of covariance function determines the smoothness and complexity of the resulting regression model.

GP has also been used in cardiovascular modelling [43–45] as a machine learning tool for various regression tasks. It is particularly useful when dealing with small to moderate-sized datasets, where it can capture complex patterns and provide uncertainty estimates in predictions. The hyperparameters in GPR can be broadly categorized into two types: kernel hyperparameters and regularization hyperparameters.

1) Kernel Function: The choice of the kernel function, also known as the covariance function or radial basis function, determines how the covariance between data points is computed. Common kernels include the radial basis function (RBF or squared exponential), Matérn, polynomial, and more.

2) Regularization Hyperparameters (Alpha): In GPR implementations, regularization hyperparameters may be available to prevent overfitting and improve model generalization. These hyperparameters control the trade-off between fitting the training data and simplifying the model.

The kernel functions in this work are set to Rational Quadratic and Matern52. In addition, the alpha range is selected to be: [0.01, 0.1, 1].

### 4) TREE

Decision Tree Regression is a supervised machine-learning technique used for regression tasks. Decision tree regression predicts continuous numerical values unlike decision trees for classification, which output discrete class labels. The core idea behind decision tree regression is to partition the feature space into regions and assign a constant value to each region, which predicts any input falling into that region.

The algorithm begins by partitioning the input data into subsets based on the values of the input features. It selects the feature and the corresponding threshold that best splits the data, aiming to minimize the variance (or other suitable loss function) of the target values within each partition. The selection is based on the chosen feature and threshold at each internal node of the tree (the decision node) [46]. The algorithm determines if the feature value of the input data point exceeds or falls short of the threshold. Depending on the result, the algorithm moves to the left or right child node.

Each subset is subjected to data partitioning and decision-making, resulting in a hierarchical structure of decision and leaf nodes. The target variable is given a prediction value when the algorithm reaches a leaf node (terminal node). The mean or median of the goal values for that location serves as this prediction value. Based on the feature values of the input, the algorithm moves through the tree from the root node to a particular leaf node to forecast a new input sample. The model’s output for that input is the predicted value at the leaf node.

The interpretability of Decision Tree Regression is its main benefit because the output tree can be inspected and understood immediately. It is robust against outliers and can handle numerical and categorical data, making it widely used in heart disease modelling and prediction [47]. However, obtaining the best performance and reliable generalisation requires rigorous hyperparameter altering and countermeasures against overfitting. In the context of tuning decision tree hyperparameters, several key parameters are considered, focusing on controlling the tree’s complexity and potential for overfitting. These hyperparameters include but are not limited to maximum depth, minimum samples split, minimum samples leaf, and maximum leaf nodes. During the hyperparameter tuning process, the Min Leaf Sizes are set to: [2, 4, 6, 8, 10], and the Surrogate Options are chosen from the set: [off, on]

The best SVR hyperparameter combination was C = 14 and a linear kernel type. A high C value indicates that the SVR model has a low tolerance for errors in the training data. In other words, the model aims to fit the training data as closely as possible, even if it means allowing more margin violations. Using a linear kernel, the SVR model attempts to draw a hyperplane in the feature space that best fits the data points while maximizing the margin between the positive and negative support vectors. This simplicity and interpretability of the linear model make it advantageous in scenarios where the relationship between the input features and the target variable can be adequately approximated by a linear function [48]. For XGBoost, the best performance was achieved with a Learning Rate of 0.1, a Maximum Depth of 3, and a Gamma value of 0.01. A moderate Learning Rate, a shallow Maximum Depth, and a conservative Gamma value indicate a well-balanced XGBoost model. It leverages a cautious approach in building trees to avoid overfitting while still allowing the model to capture relevant data patterns in the data effectively [49]. This hyperparameter combination will likely produce a robust and accurate XGBoost model, providing reliable estimates of LVEF levels in heart failure patients across distinct categories (HFpEF, HFmEF, and HFrEF).

In the case of GPR, the best performance was observed using the kernel function of rational quadratic and an alpha of 0.1. The rational quadratic kernel combines the characteristics of both the squared exponential kernel and the Matérn kernel. Matérn kernel is a versatile covariance function that can adapt to various levels of smoothness in data and provides a balance between the squared exponential kernel and the Gaussian kernel. The rational quadratic kernel is more flexible than the squared exponential kernel, allowing it to capture various patterns in the data, including short-range and long-range correlations. An alpha value of 0.1 indicates that the model is giving more importance to the squared exponential term, making it more sensitive to large-scale variations [42]. This choice could be appropriate if the underlying data has smooth patterns and long-range dependencies. Finally, for the Decision Tree Regression model, the best hyperparameter combination comprised a Min Leaf Size of 4 and ’off’ for the Surrogate Option. Choosing a minimum leaf size of 4 strikes a balance between capturing some of the complexity in the data while preventing excessive overfitting [50]. This choice suggests that the data might have sufficient complexity to warrant more splits but still benefits from regularization to control overfitting. Our results indicate that each regression model exhibited exceptional performance when tuned with its best hyperparameter combination.

### Training and testing configuration

This study introduces a nested loops framework (Fig 1), which synergistically combines leave-one-out cross-validation (LOOCV) as the outer loop and 5-fold cross-validation as the inner loop. This carefully designed approach is advocated for its ability to offer a robust and comprehensive methodology for model evaluation and hyperparameter tuning. In the outer loop, each data point is iteratively withheld as a validation set while the model is trained on the remaining data instances. This process is repeated for all data points, allowing comprehensive evaluation across various training and validation scenarios to obtain reliable performance estimates. Within the outer loop, the inner loop performs 5-fold cross-validation, partitioning the data into five subsets. The model is trained on four subsets while the remaining one is validated. This cycle is repeated five times, robustly assessing the model’s performance. The average performance metrics from these iterations are then utilized to evaluate the model’s effectiveness more comprehensively.

To optimize the model’s hyperparameters efficiently, the nested loop approach incorporates grid search technology. Grid search systematically explores a predefined hyperparameter grid, covering various combinations of hyperparameter values. At each iteration of the nested loops, the model’s performance is evaluated based on different hyperparameter configurations. This allows for an exhaustive search for the optimal set of hyperparameters that maximizes the model’s accuracy and generalization capabilities.

Using nested loops with LOOCV, 5-fold cross-validation, and grid search technique provides a rigorous and reliable framework for regression tasks in machine learning. It enables meticulous hyperparameter tuning, ensures robust model evaluation, and ultimately leads to the selection of the best-performing regression model with enhanced predictive capabilities of the LVEF estimation and increased applicability to heart failure diagnosis.

The evaluation of the regression model’s performance using key metrics, including the average Root Mean Square Error (RMSE), Mean Absolute Error (MAE), and correlation analysis, were computed. Furthermore, the estimation process was visualized through Bland-Altman plots and correlation plots [48, 51], comprehensively analysing the model’s predictive capabilities.

Furthermore, the feature importance metric quantifies the impact of each feature on reducing the impurity (e.g., RMSE) in the model. The more a feature contributes to reducing the impurity during tree construction, the more important it is considered. This importance is typically computed as the total reduction in impurity achieved by each feature over all nodes in the tree. In nested cross-validation (LOOCV and 5-fold cross-validation), the feature importance is calculated iteratively over multiple folds or model training and evaluation iterations. This ensures robustness in estimating feature importance and helps mitigate overfitting issues.

## Results

The single GeForce NVIDIA MX350 (2 GB) memory, Intel Core i5- 1135G7 (11th Gen) used to train and validate the models hourly. The training and testing phases took 10–20 minutes per model due to following the nested loop scheme. After tuning each model, the optimal hyperparameter combinations for each model are shown in Table 3.

**Table 3:** Regression models and hyperparameters tuning.

Regression Model	Hyperparameters	Best Combination
**SVM**	C Values: [0.1, 1, 10,14] Kernel Types: [linear, polynomial, rbf].	C Value = 14 Kernel Type = linear
**XGBOOST**	Learning Rate: [0.1, 0.2] Maximum Depth: [3, 5] Gamma: [0, 0.01]	Learning Rate = 0.1 Maximum Depth = 3 Gamma = 0.01
**GPR**	Kernel Functions: [rational quadratic, matern52] Alpha Range: [0.01, 0.1, 1]	Kernel Function = rational quadratic Alpha Range = 0.1
**TREE**	Min Leaf Sizes: [2, 4, 6, 8, 10] Surrogate Options:[off, on]	Min Leaf Size = 4 Surrogate Option = off

All models were trained using the extracted ECG features (TP, QTc, PR, ST-T, QRS, Mean-Entropy, and Mean-Infrequency), Table 4 summarizes the values of these features for the three groups (HFpEF, HFmEF, and HFrEF) with statistical comparisons. Table 5 shows the average RMSE values for the estimated LVEF levels, which vary over 24 hours and across different regression models with the best hyperparameters combination. In this table, each column corresponds to a specific time throughout the 24-hour cycle, and each row stands for a different regression model with the best hyperparameters combination. The RMSE values indicate the average magnitude of the errors between the estimated LVEF levels and the actual values for each period and model. Lower RMSE values show better model performance, indicating smaller errors and more accurate predictions. The table provides an overview of how the performance of the different regression models varies across the 24 hours. It allows for comparing different models and their suitability for estimating LVEF levels at contrasting times of the day.

**Table 4:** ECG features for the three groups (HFpEF, HFmEF, and HFrEF).

ECG Feature (±SD)	HFpEF	HFmEF	HFrEF	p-Value
**TP-Interval (ms)**	198±23.5	200±31.3	276±35.2	0.0014
**QTc-Interval (ms)**	451±54.1	456±44.2	501±33.7	**<0.0001**
**ST-T Interval (ms)**	302±53.6	251±46.7	248±38.9	0.0007
**QRS-Interval (ms)**	96±18.3	102±31.1	117±28.5	**<0.0001**
**Mean-Entropy**	0.73±0.022	0.83±0.042	0.76±0.036	0.0015
**Mean-InsFreq**	38.4±8.7	36.1±10.5	36.3±11.2	0.0031

**Table 5:** Average RMSE values through 24-Hours, using regression models with best hyperparameters.

Regression Model	RMSE																							
	00:01	01:02	02:03	03:04	04:05	05:06	06:07	07:08	08:09	09:10	10:11	11:12	12:13	13:14	14:15	15:16	16:17	17:18	18:19	19:20	20:21	21:22	22:23	23:24
**SVM**	**4.2**	**4.5**	**5.2**	**4.8**	**4.6**	**5.8**	**4.9**	**4.7**	**4.8**	**5.0**	**4.6**	**5.3**	**4.7**	**5.0**	**4.3**	**4.7**	**4.4**	**4.9**	**5.1**	**4.4**	**4.7**	**4.7**	**4.5**	**5.2**
**XGBOOST**	**4.4**	**4.3**	**5.0**	**4.4**	**4.5**	**5.6**	**4.9**	**4.6**	**4.1**	**4.9**	**4.5**	**5.2**	**4.7**	**5.0**	**4.3**	**4.8**	**4.3**	**4.9**	**5.0**	**4.2**	**4.2**	**4.3**	**4.2**	**5.0**
**GPR**	**3.9**	**4.6**	**4.4**	**5.1**	**4.1**	**4.7**	**5.4**	**5.1**	**4.2**	**5.0**	**4.9**	**4.5**	**5.1**	**4.8**	**5.3**	**4.3**	**4.9**	**4.4**	**4.7**	**4.9**	**4.3**	**4.3**	**3.8**	**4.5**
**Tree**	**3.6**	**4.4**	**4.3**	**5.0**	**4.2**	**4.5**	**5.5**	**4.9**	**4.0**	**4.7**	**4.9**	**4.9**	**5.5**	**4.8**	**5.1**	**5.7**	**4.8**	**4.3**	**4.9**	**4.9**	**4.2**	**4.2**	**3.4**	**4.3**

Yellow shading stands for the lowest RMSE value

**Fig 2:**
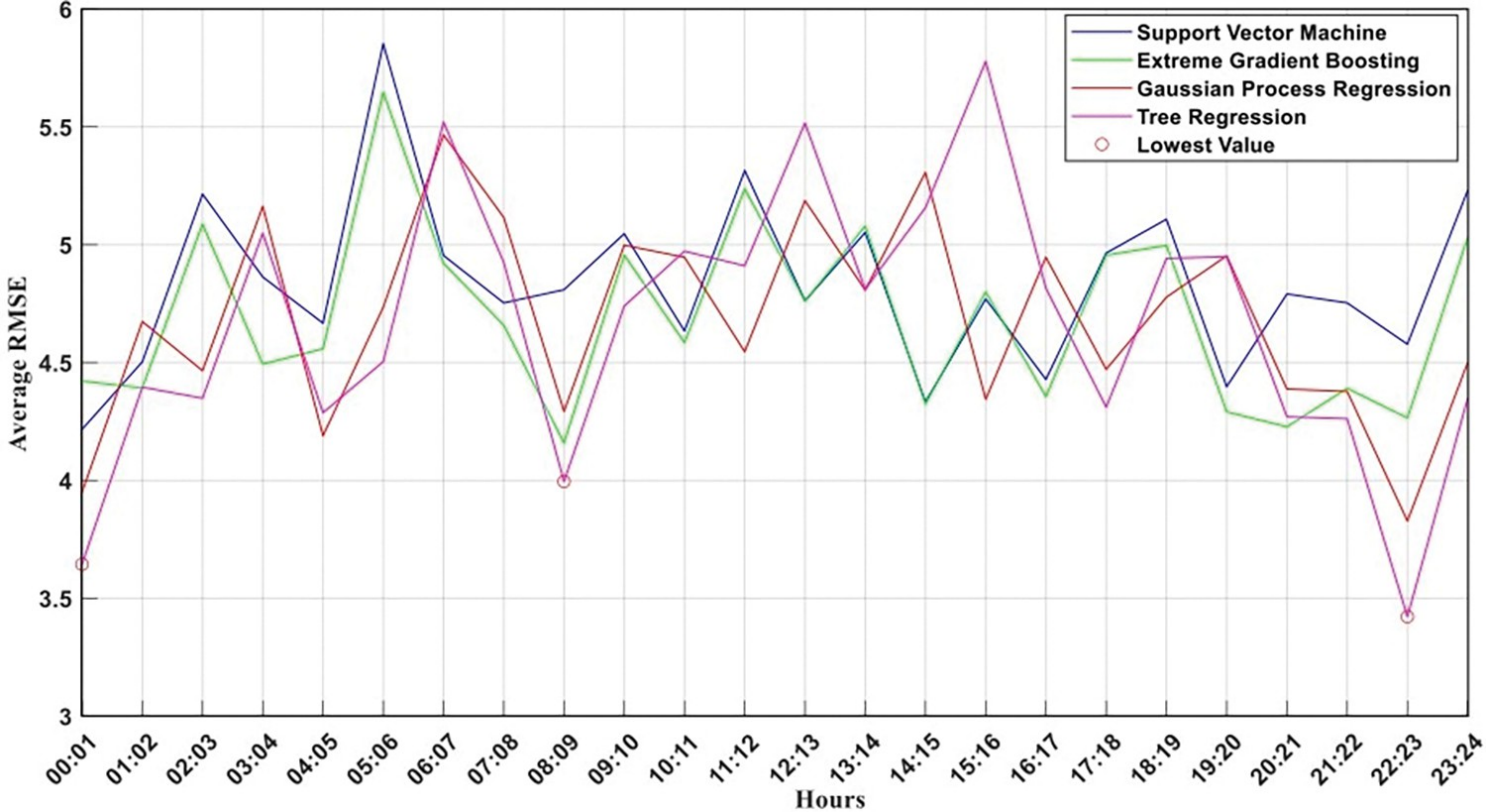
RMSE per hour using the best hyperparameters combination for the four regression models. Red circles show the hours of occurrence of the lowest RMSE values. Where QTc and QRS are found to be the most important features for evaluating the LVEF levels.

The lowest error (RMSE = 3.4) occurred between 10 pm to 11pm using the Tree function, where the minimum leaf size is 4, and the surrogate way is set to be off. While the GPR function led to an RMSE value of 3.8 at this time interval, with rational quadratic kernel function and alpha = 0.1. The XGBOOST best performance occurs in the morning interval (8–9 am) with an RMSE value of 4.1. The early morning time (12–1 am) results in the lowest error using the SVM model with a linear function and C value of 14. In addition, Fig 2 depicts the RMSE distribution across the 24 hours using the four regression models. In the latter, the time interval of the lowest possible RMSE values is highlighted in red, where the Tree regression model outperforms the other models in the three-time intervals.

**Fig 3:**
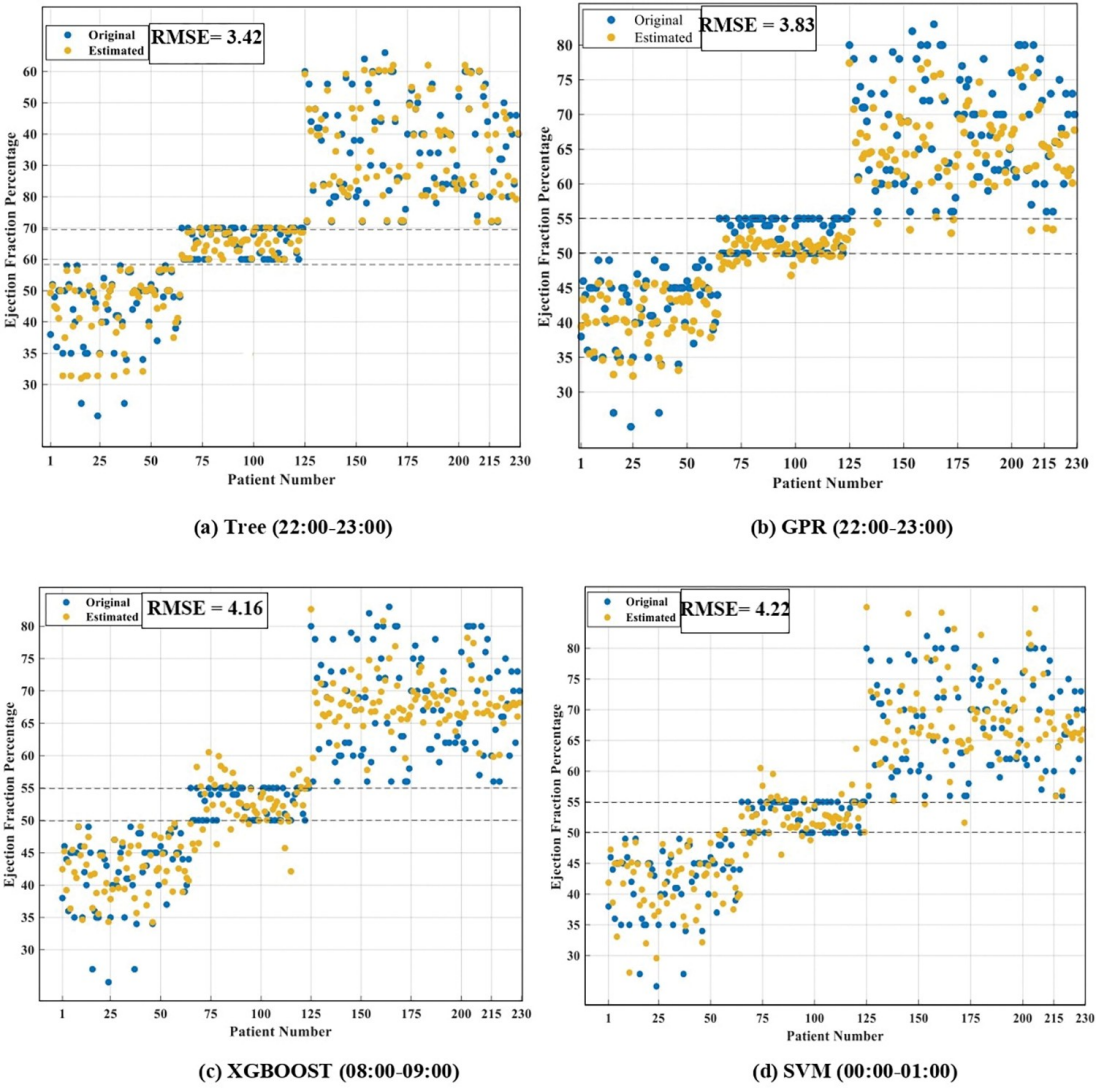
Original and estimated LVEF values per patient for hours (22:00–23:00, 08:00–09:00, 00:00–01:00) using the Tree, GPR, XGBOOST and SVM, respectively.

**Fig 4:**
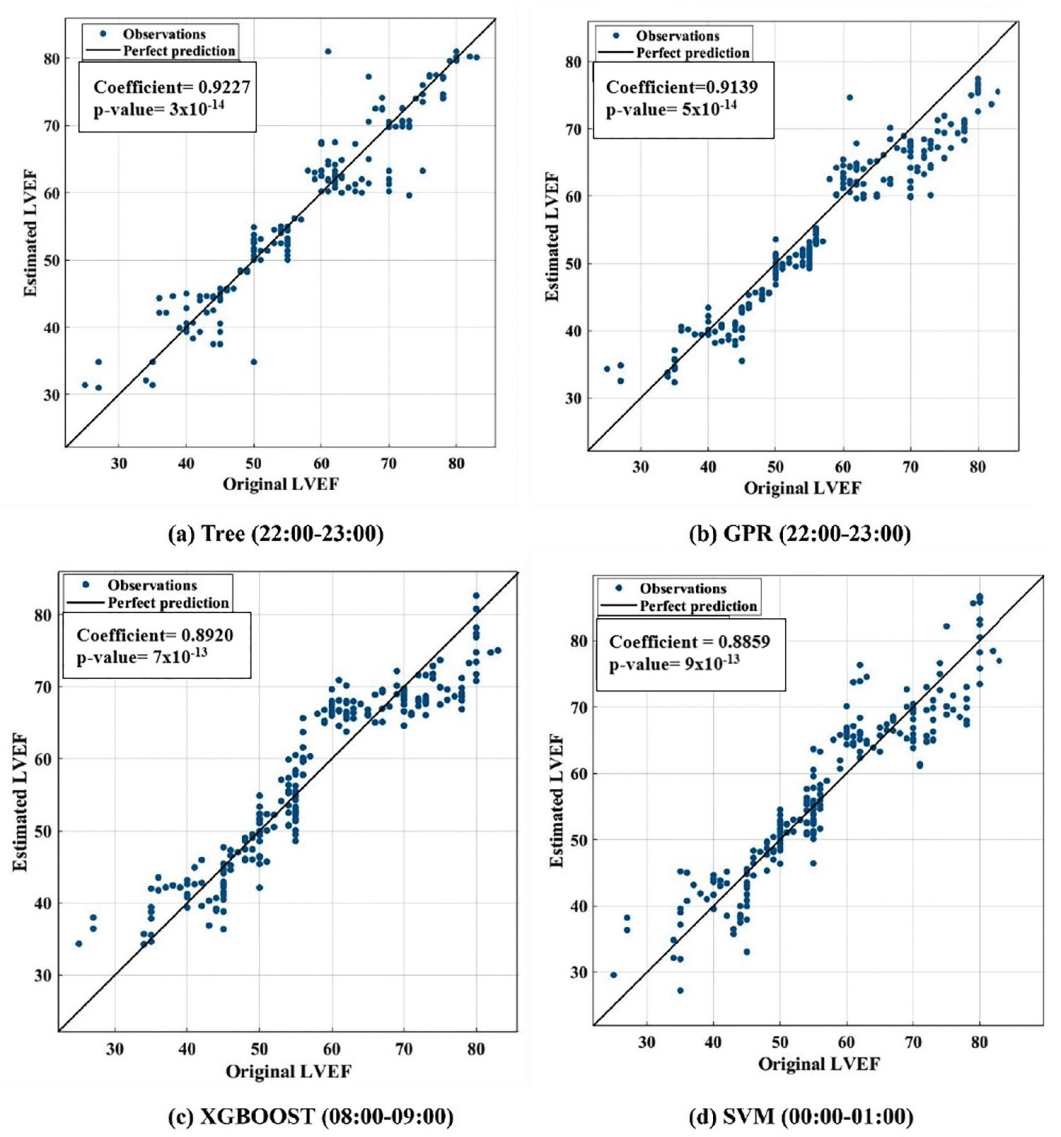
Correlation plots between the original and estimated LVEF values for hours (22:00–23:00, 08:00–09:00, 00:00–01:00) using the Tree, GPR, XGBOOST and SVM, respectively.

**Fig 5:**
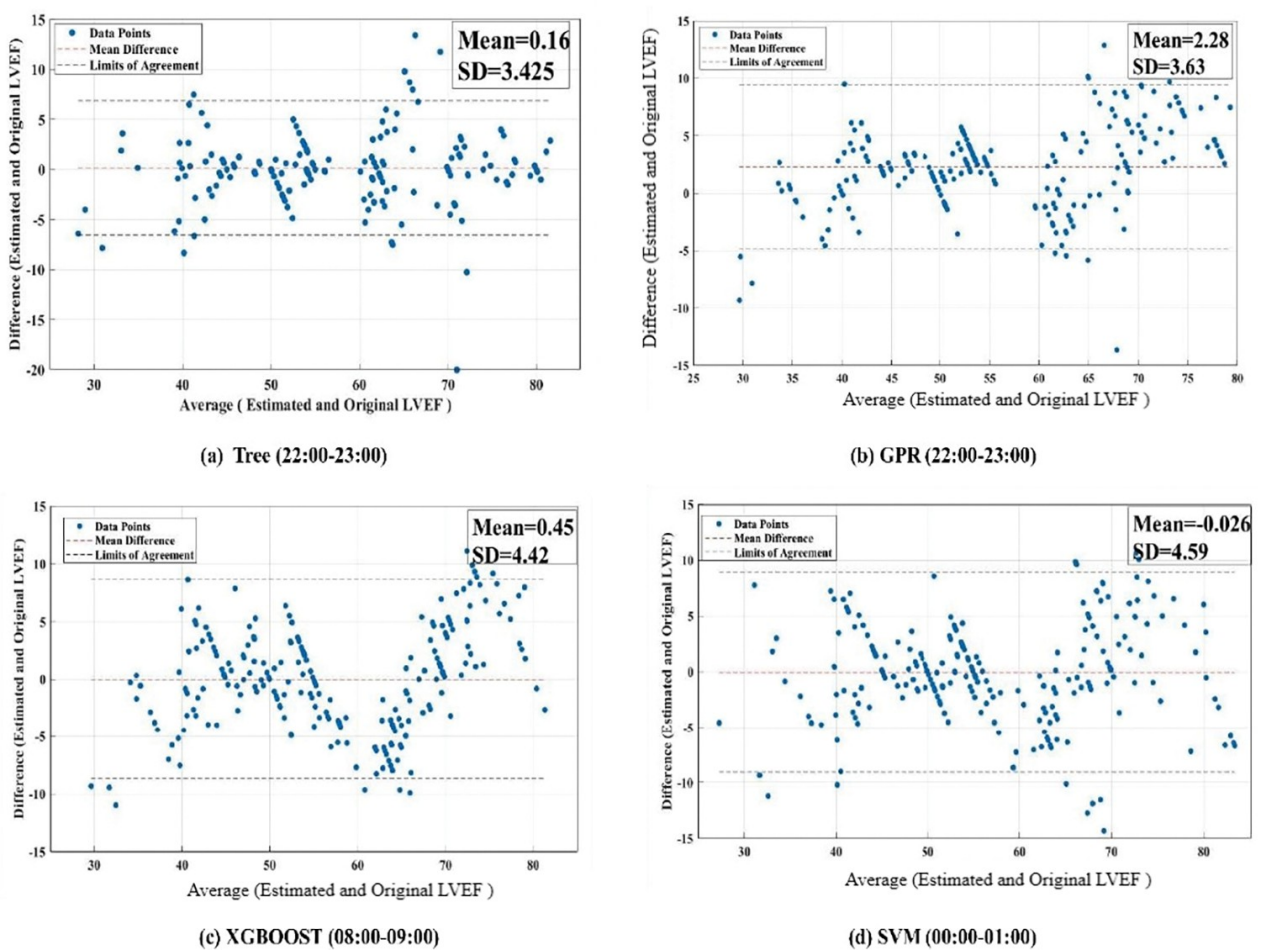
Bland-Altman plots between the average value of (Original and estimated LVEF) and their corresponding difference for hours (22:00–23:00, 08:00–09:00, 00:00–01:00) using the Tree, GPR, XGBOOST and SVM, respectively.

Furthermore, Fig 3 shows the original and estimated LVEF values for all patients at 00:00–01:00 h, 08:00–09:00 h, and 22:00–23:00 h with the lowest RMSE values. The dashed lines represent the ranges of the heart failure patients with reduced, midrange, and preserved LVEF according to ASE/EACVI guidelines. To elaborate more on the prediction models, Figs 4 and 5 show the correlation and Bland-Altman plots, respectively. The highest correlation coefficient occurred at 10–11 pm using the Tree model with 0.9227, while the correlation at 10–11 pm and 8–9 am was 0.9139 and 0.8920 using the GPR and XGBOOST, respectively. And the least correlation was from midnight to 1 am, using the SVM model. Furthermore, the mean bias was overestimated at 10–11 pm and 8–9 am while underestimated between midnight and 1 am.

**Fig 6:**
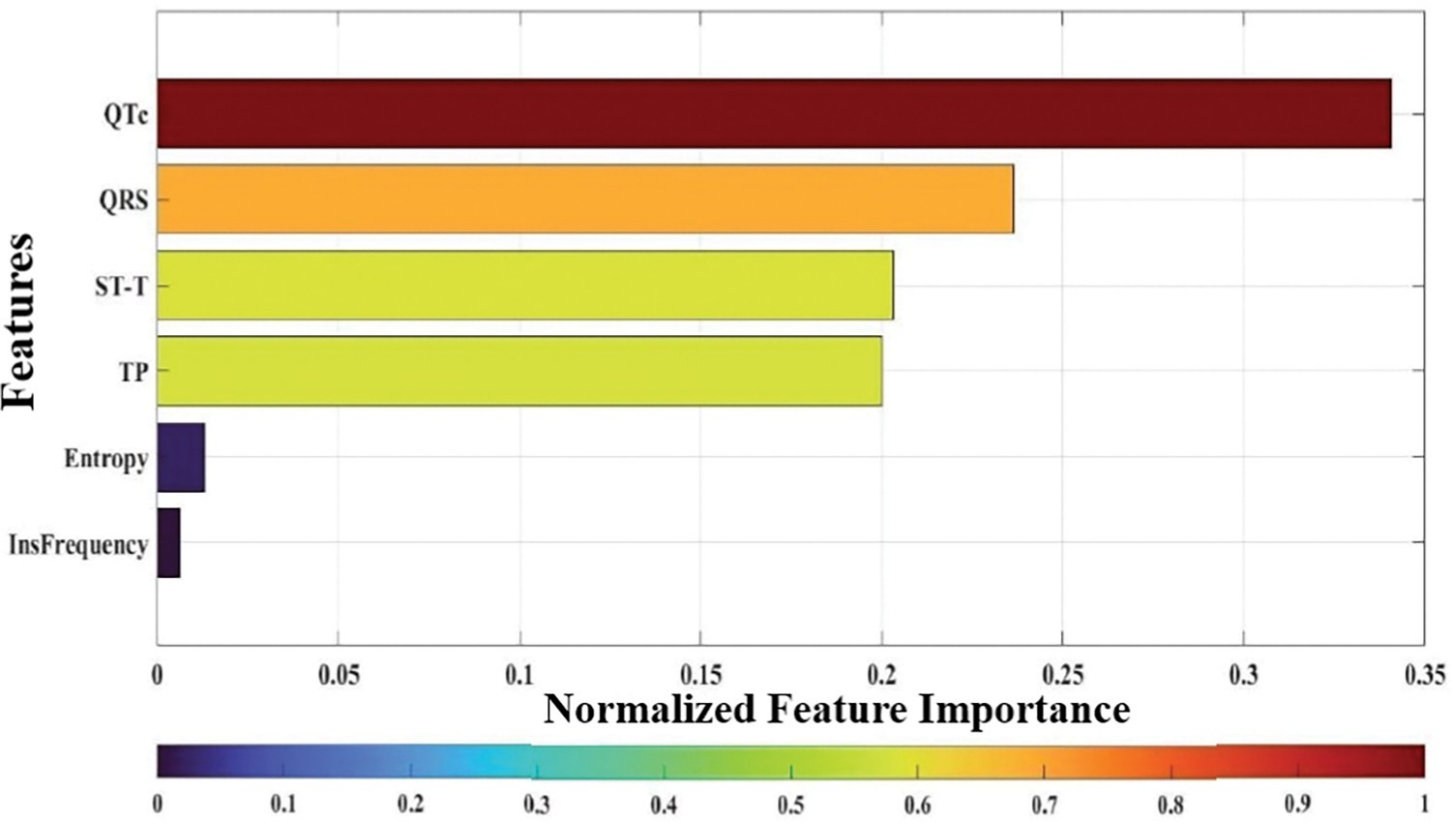
Feature importance over the Tree regression model (10:00–11:00pm). This study used normalized importance scores for the ECG features (QTc, QRS, ST-T, TP, Entropy, and Instant Frequency) to estimate the LVEF levels in the three HF categories. Indicating that QTc is the most important feature for evaluating the LVEF levels.

Moreover, in this study, we have evaluated the feature importance using the best regression model. The results are presented in Fig 6, highlighting the relative significance of each feature in contributing to the model’s predictive performance. The feature importance values have been calculated based on their influence on the target variable, providing valuable insights into the key determinants driving the LVEF estimation. This analysis allows us to identify the most influential features, enabling a deeper understanding of the underlying factors influencing the outcome of the regression model. The comprehensive evaluation of feature importance aids in interpreting the model’s behaviour and helps informed decision-making for our research application.

**Fig 7:**
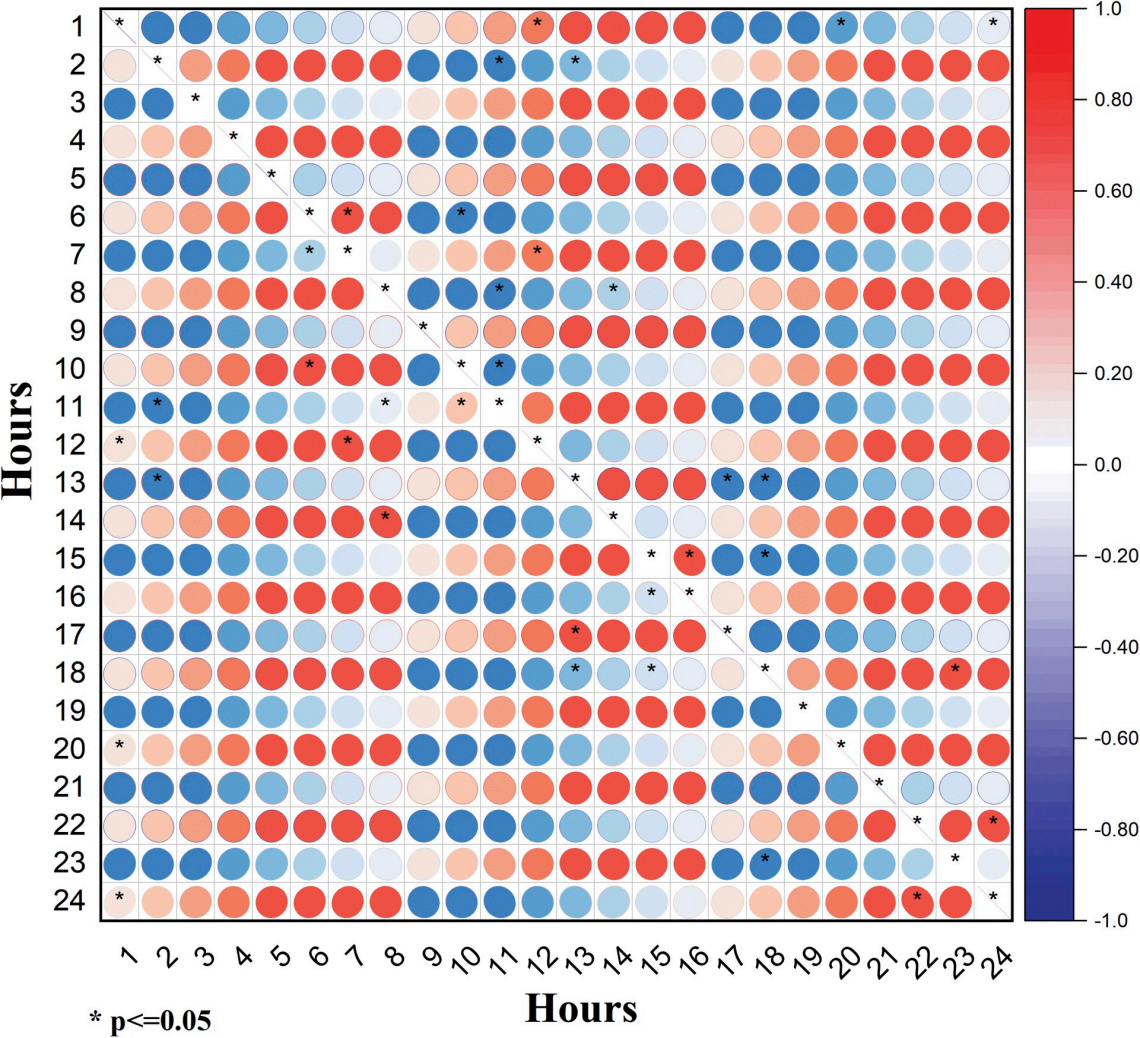
Correlation matrix of the RMSE values through 24-Hours, using the best regression model. Black asterisks show the hours of statistical significance to circadian changes.

Furthermore, analyzing RMSE values through the 24 hours allows us to assess how sensitive the models are to circadian changes at different times of the day. If the RMSE values exhibit significant fluctuations or patterns, it suggests that the model is sensitive to circadian changes. As shown in Fig 7, the p-values in the correlation matrix indicate the statistical significance of the observed correlations using the best regression model. A low p-value suggests that the correlations are unlikely to be due to random chance, reinforcing the validity of the relationships. Whereas, the model is sensitive to circadian changes when moving from morning to afternoon hours (e.g., at 1–12 h, 2–13 h, 7–12 h and 8–14 h). Also, this analysis demonstrates the model’s ability to capture circadian changes at the afternoon intervals (e.g., 13–17 h, 13–18 h, and 15–18 h), and at the evening intervals (e.g., 18–23 h, and 22–24 h).

## Discussion

This study provides valuable insights into the importance of utilizing ECG features to estimate LVEF levels in heart failure patients categorized into HFpEF, HFmEF, and HFrEF groups. Through a comprehensive investigation, the study identifies the significance of specific ECG features in accurately predicting LVEF levels throughout a 24-hour study.

By employing regression analysis, the study focuses on minimizing the RMSE to achieve the best possible predictive performance. By identifying the optimal hyperparameters and model configurations, the study successfully achieves high accuracy in each of the four regression schemes at various hours in the heart’s circadian rhythm. This remarkable performance demonstrates the potential of ECG features integrated into machine learning algorithms as a valuable assistive screening tool for heart failure diagnosis and treatment These ECG parameters, such as QRS duration, ST segment changes, and T-wave abnormalities, closely mirror the temporal aspects of ventricular systole and diastole. The QTc interval, representative of ventricular repolarization, intricately mirrors the duration required for myocardial cells to recover their resting state following depolarization. As LVEF signifies the efficacy of ventricular contraction, its prediction is inherently linked to the temporal aspects of cardiac repolarization reflected in the QTc interval. Similarly, the QRS duration reflects the propagation of electrical impulses during ventricular depolarization, intricately tied to mechanical events. Changes in QRS duration can be indicative of altered ventricular conduction, which in turn influences the synchronization of contraction, affecting LVEF. Thus, by harnessing the electrophysiological nuances encoded in ECG features, the models capitalize on the profound connection between electrical signalling and mechanical performance. This synergy offers a more comprehensive understanding of cardiac function, rendering the models adept at predicting LVEF with heightened accuracy, drawing from the intricate dialogue between electrophysiological dynamics and mechanical behaviours.

Compared to previous work (Table 6), SVR models were employed to predict LVEF using heart rate variability (HRV) data derived from 24-hour ECG recordings of 92 patients participating in the IDEAL study [52]. The patients were categorized based on their LVEF as preserved, mid-range, or reduced. The SVR models exhibited varying performance throughout the 24 hours. The most accurate estimation, with an RMSE of 10.4, was achieved during the time interval between 6 pm and 7 pm. The polynomial kernel function was employed for this interval, and the model incorporated 18 of the 25 available features. In another study [53], three different models, namely SVM with RBF kernel, Generalized Linear Model (GLM), and CNN (Convolutional Neural Network), were employed to estimate the LVEF in heart failure patients belonging to the three distinct categories. The study used clinical profiles from 303 patients with CAD acquired from American and Greek patient databases. The goal was to compare the performance of the three models in predicting LVEF in these patients. After rigorous evaluation, it was observed that the CNN model surpassed the other approaches with an RMSE of 4.13 and a correlation coefficient of 0.85.

**Table 6:** Summary of the studies for LVEF estimation in preserved, midrange, and reduced HF patients. Best performance highlighted in yellow.

Author (Year)	ECG Signal Duration	Data	Method/Features	Regression	RMSE	Correlation Coefficient	Bland-Altman Mean ± 2 Std	MAE
**^**54**^**	**1hour (24h study)**	**92 (American Cohort)**	**HRV features**	**SVR**	**10.4**	**0.61**	**0.53±20.44**	**NA**
**^**55**^**	**NA**	**303 (American, Greek Cohorts)**	**Clinical profiles**	**CNN**	**4.13**	**0.85**	**0.39 ± 11.61**	**NA**
**Current study**	**1hour (24h study)**	**229 (American, Greek Cohorts)**	**ECG features**	**SVR** **XGBOOST** **GPR** Tree	**4.22** **4.16** **3.83** 3.42	**0.89** **0.89** **0.91** 0.92	**-0.03±9.18** **0.45±8.84** **2.28±7.26** 0.16±6.85	**3.13** **3.24** **2.94** 2.28

The present study surpasses the performance of previous works [52–54] in the literature for estimating LVEF levels for heart failure patients across the three distinct categories. The results of this investigation reveal that the optimal performance is achieved during the time interval between 10 pm and 11 pm, using a tree-based model. This model exhibits an impressive RMSE of 3.42, a high correlation coefficient of 0.92, and a Bland-Altman analysis demonstrating a mean difference of 0.16 with a narrow limit of agreement of ±6.85%. Furthermore, the MAE is remarkably low at 2.28%, meaning that the model’s predictions are closer to the actual values.

### Clinical relevance and ECG features importance

ECG features obtained through ambulatory Holter monitoring have been established as valuable predictors of total mortality and the progression of heart failure. This continuous monitoring technique offers unique insights into a patient’s cardiac health, allowing for a comprehensive evaluation of heart rhythm and electrical activity over an extended period. Even when accounting for other known risk factors, such as age, gender, and comorbidities, the information derived from Holter monitoring provides additional and valuable prognostic information. However, estimating LVEF in heart failure patients hourly using ECG features can offer valuable insights into the temporal patterns and fluctuations of cardiac function. This hourly analysis can enhance our understanding of how LVEF levels vary throughout the day, potentially identifying when LVEF estimation is more accurate and best correlated with clinical variations or when heart function is at its optimal state. This understanding of temporal variation may be crucial for adjusting treatment strategies or interventions based on the patient’s circadian rhythm and physiological fluctuations. This work demonstrates superior performance during the evening (22–23) and early morning hours (12 am-1 and 8–9) for estimating LVEF levels. Additionally, these hourly intervals align with previously reported high-risk periods for increased mortality and myocardial infarcts observed in the early morning and evening hours [55–57].

Furthermore, using the most effective regression model, we evaluated feature importance during this study. Our findings revealed that the most crucial features contributing to LVEF prediction are QTc, QRS, ST, and TP in descending order of importance. These specific ECG parameters significantly influenced the target variable, supplying valuable insights in characterizing cardiac function and highlighting their potential as important clinical indicators in assessing heart failure patients.

The prominence of QTc as the most important feature can be attributed to several key factors [58–61], QTc interval stands for the duration of ventricular depolarization and repolarization, encompassing the electrical conduction time within the heart. In HF patients, electrical conduction abnormalities are common due to ventricular remodelling and changes in ion channel kinetics. Prolonged QTc intervals may show delayed repolarization, which can be associated with impaired ventricle (which involves alterations in ventricular size, shape, and function) and reduced LVEF. HF patients are at increased risk of arrhythmias, and QTc prolongation is a known risk factor for life-threatening ventricular arrhythmias, abnormal QTc intervals are indicative of arrhythmogenic substrate and can influence LVEF by potentially triggering life-threatening arrhythmias that may affect cardiac function. In addition, QTc interval is influenced by autonomic nervous system activity, specifically parasympathetic and sympathetic inputs to the heart. HF patients often have altered autonomic regulation, and QTc prolongation may be linked to autonomic dysfunction, which can affect cardiac function and LVEF. Some medications commonly used in HF management can affect the QTc interval. The QTc-prolonging effects of certain drugs may contribute to the importance of this feature in the model, as medication use is an essential consideration in LVEF estimation.

Several studies have shown that QRS duration is a prognostic marker in HF patients [62–64]. The QRS complex is the ventricles’ electrical activation and later mechanical contraction. In HF patients, mechanical desynchrony is a common phenomenon, where different regions of the ventricles contract uncoordinated. QRS prolongation can indicate bundle branch blocks (BBBs). These conduction abnormalities can affect the ventricular function and contribute to reduced LVEF [65]. In addition, for patients undergoing cardiac resynchronization therapy (CRT), QRS duration is a critical parameter for determining eligibility for CRT implantation. CRT effectively improves mechanical synchrony in HF patients with electrical desynchrony and prolonged QRS duration. By considering QRS duration in LVEF prediction, clinicians can gain insights into potential CRT candidacy and tailor treatment options for better patient outcomes.

Moreover, the STT interval is closely related to T-wave morphology. T-wave abnormalities, such as inversion or flattening, are commonly seen in heart failure patients [66–68]. These T-wave changes can indicate valuable information about myocardial ischemia or injury and can contribute to LVEF prediction accuracy. Variations in the TP interval may show abnormalities in repolarization, which can be linked to impaired cardiac function in HF patients. Those alterations have been associated with an increased risk of arrhythmias, such as ventricular tachycardia and ventricular fibrillation. Several studies have reported a correlation between abnormal TP intervals and reduced LVEF in HF patients. Prolonged TP intervals have been associated with impaired ventricular function, while shortened TP intervals may show myocardial remodelling and potential deterioration of cardiac performance.

The prioritization of these specific features based on their impact on the LVEF estimation reinforces the model’s ability to discern critical patterns and relationships among various ECG parameters. This knowledge is instrumental in enhancing our understanding of the underlying physiological mechanisms that govern cardiac health and dysfunction.

The proposed technology, which leverages regression models based on circadian ECG features, has the potential to serve as a valuable tool for early HF diagnosis and risk assessment. A more detailed explanation of its practical applications and requirements for implementation:

1. Data collection and Integration:

• Data sources: Access to ECG data is a fundamental requirement. This data can be collected from various sources, including hospitals, clinics, and ambulatory ECG monitors.

• EHR Integration: Integration with electronic health records (EHR) systems is essential for streamlined data access and patient history analysis. This enables healthcare providers to have a comprehensive view of the patient’s health status.

2. Machine learning infrastructure:

• Training and validation: Rigorous model training and validation, using large and diverse datasets, are critical to ensure the reliability and accuracy of the technology.

3. Clinical validation:

• Collaboration with healthcare professionals: Close collaboration with cardiologists and other healthcare professionals is crucial for the clinical validation of the technology. Their expertise ensures that the technology aligns with established medical practices and standards.

• Clinical trials: Conducting clinical trials to assess the technology’s performance in real-world healthcare settings is a key component. This helps establish its clinical efficacy and safety.

4. Regulatory compliance:

• Quality assurance: Implementing quality assurance protocols and adhering to Good Clinical Practice (GCP) and Good Manufacturing Practice (GMP) standards are important for maintaining high-quality healthcare technology.

5. Privacy and security:

• Data protection: Safeguarding patient data is paramount. Adherence to data protection regulations, such as Health Insurance Portability and Accountability Act (HIPAA) in the United States or General Data Protection Regulation (GDPR) in the European Union, is mandatory.

• Security measures: Implementing robust security measures to protect patient data from breaches and unauthorized access is essential.

6. Clinical workflow:

• Integration into ECG testing: Our technology can be seamlessly integrated into the clinical workflow as a supplementary tool during routine ECG testing.

• Automated LVEF estimation: When an ECG is performed, the technology can automatically generate an LVEF estimation, which is then made available to the attending physician.

• Physician assessment: Physicians can use this information to identify patients at risk for HF and initiate further diagnostic tests or interventions as necessary.

In conclusion, implementation in medical practice involves adherence to specific protocols related to data collection, machine learning infrastructure, clinical validation, regulatory compliance, data privacy, and a secure clinical workflow. These protocols are vital to ensure the accurate and ethical application of this technology for the benefit of patients and healthcare providers.

## Limitations and future work

Despite the effectiveness of machine learning-based models in predicting LVEF, our study has identified several limitations associated with these models. First, we focused on using six features extracted from the circadian ECG for LVEF prediction. Nevertheless, there is a need for further exploration and investigation of more features, such as QRS area, the amplitude of peak changes, and the association with EDR (ECG-derived respiration), to gain better insights into their impact on LVEF predictions. Furthermore, while the current study used a dataset including patients from both American and Greek populations, it is essential to subject the trained models to further testing on more diverse patient groups to ensure broader applicability and generalization of their performance. In this study, the patient cohort consists of a significantly higher proportion of male participants when compared to females; future studies may apply additional investigations of LVEF prediction on a more evenly distributed dataset between male and female subjects. In future research, the regression models developed in this study may have the potential to be adapted or extended to predict various parameters related to heart health (e.g., myocardial ischemia), their applicability to underlying diseases that can lead to heart failure would require additional research and validation.

## Conclusion

This study highlights the potential of ECG features derived from 24-hour recordings as a promising alternative to the current echocardiography gold standard for finding LVEF levels in CAD patients. From a machine learning perspective, the developed approach offers valuable insights into finding ECG patterns suitable for accurately estimating LVEF in different heart failure categories (preserved, midrange, and reduced).

Furthermore, the proposed study aims to extend the application of LVEF assessment to communities where access to the required instruments is limited due to economic challenges or lack of clinical expertise. The study’s outcomes hold the potential to aid the development of a model to predict the HF phenotype or track its changes during therapy, providing a versatile tool for exploring disease pathophysiology and objectively assessing therapeutic approaches in future HF patients.

Notably, the proposed machine learning model offers simplicity, efficiency, and cost-effectiveness, enabling continuous cardiac analysis and representing a viable alternative to more elaborated gold standard techniques. As research advances, using more extensive datasets and leveraging deep learning techniques will help compare the proposed model’s performance to other methods. Overall, this research contributes to advancing the field of ECG-based LVEF estimation and its potential applicability, particularly in resource-constrained settings.
